# Mesoporous bioactive glass nanoparticles doped with magnesium: drug delivery and acellular *in vitro* bioactivity

**DOI:** 10.1039/c9ra01133a

**Published:** 2019-04-17

**Authors:** Zakaria Tabia, Khalil El Mabrouk, Meriame Bricha, Khalid Nouneh

**Affiliations:** Euromed Research Center, Euromed Engineering Faculty, Euromed University of Fes, Eco-Campus Meknes Road, Campus UEMF, BP51 30 030 Fes Morocco k.elmabrouk@ueuromed.org +212 537 716 040 +212 662 054 920; Laboratory of Physics of Condensed Matter (LPMC), Department of Physics, Ibn Tofail University Kenitra Morocco

## Abstract

The effects of the magnesium doping of binary glass (Si–Ca) on particle texture, on the biomineralization process in simulated body fluid (SBF) as well as on drug loading and release were examined. For this purpose, magnesium-doped binary bioglass nanoparticles (85SiO_2_–(15 − *x*)CaO–*x*MgO, with *x* = 1, 3, 5 and 10 mol%) were prepared by a base catalysed sol–gel method. N_2_ adsorption isotherm analysis showed an enhancement in specific surface area as the Mg molar fraction increased. In addition, the FTIR spectra of the samples after soaking in SBF for various periods of time confirmed the presence of new chemical bonds related to the apatite phase, as was also confirmed by SEM observations. XRD patterns of the samples after soaking revealed that the crystallization to form a more stable apatite-like phase was hindered with increasing magnesium content in the glass composition. Furthermore, it was proved that the kinetics of drug release improved with increasing magnesium content. The porosity and the specific surface area were found to be responsible for this improvement.

## Abstract 2

The effects of the magnesium doping of binary glass (Si–Ca) on particle texture, on the biomineralization process in simulated body fluid (SBF) as well as on drug loading and release were examined.

## Graphical abstract

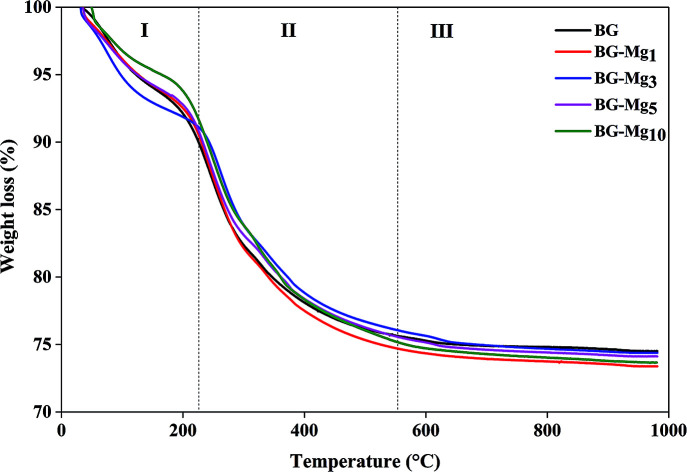

## Introduction

In the last two decades, an increasing demand for bone substitutes has been observed, due to the increase of bone diseases, traumatic injuries, and aging of the population. To fulfill this urging demand, tissue engineering and biomaterials science have been intensively studied.^1^ Among all biomaterials, ceramics and bioactive glasses have been shown to be promising candidates for bone regeneration applications. These inorganic materials are considered attractive, essentially because of their capability to bone-bond. After their implantation in a bone defect, they develop a carbonated hydroxyl apatite-like layer (CHA) on their surface, similar to the mineral phase of bone. This newly formed layer provides adhesion and strong bonding with bone tissues. Moreover, and based on various i*n vivo* studies, bioactive glasses have shown superior biological properties compared with other bioceramics,^2–4^ due to their distinct features and properties.

Since the development of the first composition by Hench *et al.* in 1969,^5^ bioactive glasses have gained a lot of attention over the years. Many studies have been carried out to develop new formulations and compositions of these bioactive materials for use in the tissue engineering field. Until now, three bioactive glass generations can be distinguished.^6^ The first generation corresponds to bioactive glasses prepared by the melt-quench method, which began with the composition reported by Hench and *et al.* in the Na_2_O–CaO–P_2_O_5_–SiO_2_ system, known as the 45S5.^5^ Then, in 1991, Li *et al.* introduced the second generation by using sol–gel chemistry to produce glasses with superior textural properties.^7^ The sol–gel method is an alternative way to prepare bioactive glasses at lower temperature. Moreover, it provides glasses with high porosity, homogeneity and purity, which are essential elements for enhancing the bioactive response.^6^ The superior textural properties allow the surface interaction with biological fluids to be increased, thus accelerating the kinetics of formation of the apatite layer both *in vitro* and *in vivo*.^8^ The third and most recent generation, which was initiated in 2004, is based on the addition of structure directing agents (surfactants) in the sol–gel chemistry to further increase the textural properties and thus accelerate the bioactive response mechanisms.^9^ These materials, referred to as template glasses, are characterized by an ordered structure of mesopores, and they show superior apatite forming ability over conventional sol–gel glasses. Their well-organized mesoporous structure and increased textural properties aid rapid ion exchange between the biological surrounding medium and the glass surface, which leads to fast supersaturation and rapid precipitation of the new apatite layer on their surface.^6,9^ Incorporation of structure directing agents has given bioactive glasses other potential fields of application as systematic delivery systems and implantable local-delivery devices, and it has enabled them to be multifunctional materials.^2,6^ In contrast to the melt-quenched method, sol–gel permits the synthesis of bioactive glasses in less complex composition systems and with high silica contents.^10^ Martinez *et al.* investigated the bioactivity of sol–gel derived binary glasses in the system SiO_2_–CaO and showed that even with a silica content up to 90% mol, the bioactivity is retained.^11^

As previously mentioned, bioactive glasses can also serve as multifunctional systems to be used not only for their bioactivity but also as a carrier for drugs, growth factors or other biomolecules. In this context, template glasses and mesoporous pure silica materials are the most commonly investigated due to their enhanced textural properties. In this sense, bioactive glass nanoparticles are of high importance. Their high surface to volume ratio make them an excellent candidate for bone regeneration. Additionally, these nanoparticles can serve as fillers in a polymeric matrix.^12,13^ Their morphological characteristics can improve their incorporation more homogeneously. Bioactive glass nanoparticles can also be used as coatings for metallic implants or for the preparation of injectable bioactive cements.^14^

Incorporation of metallic ions into the glass composition and the study of their effects on the glass properties have been the focus of many research papers in the past few years.^15–17^ This doping can give additional functionalities to the glass such as angiogenesis and antibacterial effects.^16,17^ One of the most investigated elements is magnesium. It is a trace alkali-earth metal naturally present in the human body. 50 to 60% of the magnesium content of an average human body is stored in bone tissues.^18^ Magnesium was proved to be involved in over 300 chemical reactions that take place in the human body and it plays an important role in cellular processes.^19^ For example, it can stimulate osteoblast proliferation, a process that is crucial for bone development, maintenance and repair.^20^ Additionally, Mg depletion results in impaired bone growth, increased loss in trabecular bone and bone resorption, highlighting the substantial role that Mg plays in bone metabolism.^21^ Doping bioactive glasses with magnesium has been previously investigated.^22^ For instance, Dietrich *et al.* studied the effect of magnesium doping of 46S6 on the *in vitro* bioactivity of this composition. They found that magnesium influences the formation and the evolution of the newly formed layers, and the intensity of these changes depends largely on magnesium content added in the glass matrix.^23^ Another study was performed by Riti *et al.* on the effect of synthesis route and magnesium addition on the structure and bioactivity of a sol–gel derived binary glass. The results demonstrated that MgO improves the bioactivity of glasses prepared by the alkali mediated route and inhibits it in those prepared by the acid route.^24^ Magnesium can enter the glass network as a modifier or an intermediate depending on its content and the overall glass composition.^22^ In this context, there is still some controversy on its role in the glass network and its effect on the glass properties, including bioactivity.^22^ In the present study, we shed new light on the effect of magnesium doping on the properties of bioactive glasses. Herein, we investigate the acellular *in vitro* bioactivity as well as loading and release of amoxicillin of glasses in the system 85SiO_2_–(15 − *x*)CaO–*x*MgO. The studied glasses have been produced using sol–gel chemistry with the incorporation of a structure directing agent. To the best of our knowledge, magnesium has never been studied under these circumstances. Accordingly, this paper gives additional insight on the effect of magnesium on glass bioactivity and its capacity to load and release an antibiotic agent.

## Materials and methods

### Materials

Tetraethyl orthosilicate (SiC_8_H_20_O_4_), calcium nitrate tetrahydrate (Ca(NO_3_)_2_·4H_2_O), magnesium nitrate hexahydrate (Mg(NO_3_)_2_·6H_2_O), hexadecyltrimethylammonium bromide (C_19_H_42_BrN, CTAB), ethanol (C_2_H_6_O) and ammonia hydroxide (NH_4_OH) were used to produce the bioactive glass powders. For SBF and PBS (Phosphate Buffered Saline) preparation, the following chemicals were used: calcium chloride (CaCl_2_), potassium chloride (KCl), sodium chloride (NaCl), sodium sulfate (Na_2_O_4_S), sodium bicarbonate (NaHCO_3_), sodium phosphate dibasic (Na_2_HPO_4_) magnesium chloride hexahydrate (MgCl_2_·6H_2_O), potassium phosphate dibasic trihydrate (HK_2_O_4_P·3H_2_O), Trizma (C_4_H_11_NO_3_) and hydrochloric acid (HCl). Toluene (C_7_H_8_) and APTES (H_2_N(CH_2_)_3_Si(OC_2_H_5_)_3_) were employed for surface functionalization. All chemical products mentioned above were purchased from Sigma Aldrich and were used without further purification.

### Preparation of BG-Mg_*x*_ powders

Five compositions of mesoporous bioactive glasses, as shown in Table 1, were prepared by the sol–gel method at room temperature, using hexadecyltrimethylammonium bromide (CTAB) as a structure directing agent. The synthesis was performed according to procedures described elsewhere, with slight modifications.^25,26^ Briefly, 1 g of the surfactant was dissolved in a mixture of distilled water (150 ml), ethanol (30 ml) and ammonium hydroxide (2 ml), which was used as a catalyst. After dissolving CTAB, proper amounts of calcium nitrate tetrahydrate and magnesium nitrate hexahydrate were added to the mixture depending on the glass composition, followed by continuous stirring for 30 minutes. Then, 15.6 ml of TEOS was added dropwise and the resultant solutions were stirred for four additional hours. The white precipitates were then filtered and washed with distilled water. After that, samples were dried at 80 °C for 18 hours. Finally, they were thermally heat treated at 550 °C for 6 hours using a heating rate of 1 °C min^−1^, in order to remove the remaining CTAB and nitrates.

**Table 1:** Molar compositions of the studied glasses

Sample acronym	SiO_2_ (% mol)	CaO (% mol)	MgO (% mol)	Molar composition
BG	85	15	0	85SiO_2_–15CaO
BG-Mg_1_	85	14	1	85SiO_2_–14CaO–1MgO
BG-Mg_3_	85	12	3	85SiO_2_–12CaO–3MgO
BG-Mg_5_	85	10	5	85SiO_2_–10CaO–5MgO
BG-Mg_10_	85	5	10	85SiO_2_–5CaO–10MgO

Three of our glass powders, *i.e.* BG, BG-Mg_3_ and BG-Mg_5_, were surface-functionalized with 3-aminopropyl groups by using a post-grafting procedure. Briefly, one gram of bioactive glass powder was dispersed in 100 ml of toluene by ultrasonication for 30 minutes. After that, 5 ml of APTES was added and the system was refluxed at 80 °C for 24 hours under a nitrogen atmosphere. The powder was then collected by filtration, washed with toluene and ethanol and dried at 100 °C for 18 hours. The amino functionalized glasses were designated as n-BG, n-BGMg_3_ and n-BGMg_5_.

### Characterization

The chemical composition of our powders was determined using Inductively Coupled Plasma-Atomic Emission Spectroscopy (ICP-AES), which was performed on a JobinYvon Horiba-Ultima 2 apparatus. The results were obtained using the average of three measurements.

Structural characterization was performed using X-ray Diffraction (XRD) and Fourier Transformed Infrared Spectroscopy in Attenuated Total Reflection mode (FTIR-ATR). The XRD patterns of all calcined powders were recorded using an automated X-ray powder diffractometer (PAnalytical) using CuKα radiation at a voltage and current of 45 kV and 40 mA, respectively. The data were collected in the 2*θ* range of 10–60°. FTIR-ATR analysis was conducted on bioactive glass powders using an IS50 spectrometer. The samples were analyzed in a total of 64 iterations in the range of 400 to 4000 cm^−1^ with a resolution of 4 cm^−1^. Thermogravimetric Analysis (TGA) was carried out on the dried samples using a TA Instruments Q500 thermal analyzer to determine the appropriate stabilization temperature. The scans were performed using a heating rate of 10 °C min^−1^ to a maximum temperature of 1000 °C under an air atmosphere.

Textural properties were determined from N_2_-gas adsorption/desorption analysis using a 3Flex from Micromeritics. Measurements were performed at 77.37 K. Before each measurement, samples were degassed at 100 °C for 12 hours to remove moisture and any other contaminants from the surface. Surface area was obtained by means of the Brunauer–Emmett–Teller (BET) method,^27^ whereas total pore volume was calculated from the amount of N_2_ adsorbed at a relative pressure of 0.95, corresponding to complete pore filling, and the pore size distribution was obtained using the Barrett–Joyner–Halenda (BJH) method.^28^

The morphology of the samples was examined using Scanning Electron Microscopy (SEM) under high vacuum, at an accelerated voltage between 12.5 and 15 kV after being coated with carbon.

### Bioactivity assessment

The *in vitro* bioactivity of the prepared glass samples was assessed by immersion in Simulated Body Fluid (SBF), prepared as described by Kokubo *et al.*^29^ All specimens were immersed for 3, 6, or 12 hours or 1, 3, 7, or 14 days. SBF is an ionic solution in which the concentration of each ion is similar to that of human plasma, as shown in Table 2. The test was performed using BG powders. Equal amounts of samples were incubated in SBF at a concentration of 2 mg/1 ml in clean and sterile bottles at 37 °C under continuous stirring. After each selected period, samples were removed from the SBF by filtration, washed with distilled water and dried at 60 °C for 24 hours. The phase and chemical structure of the obtained samples were then characterized using FTIR spectroscopy and XRD. For detecting the crystallite formation, the surface samples were examined by using Scanning Electron Microscopy (SEM). Furthermore, the pH value of all the filtrated solutions was measured as well.

**Table 2:** Ion concentration (mM) in SBF and human blood plasma

Ion	Na^+^	K^+^	Ca^+^	Mg^+^	Cl^−^	HCO_3_^−^	HPO_4_^2^	SO_4_^2−^
SBF	142	5	2.5	1	147.8	4.2	1	0.5
Human blood plasma	142	5	2.5	1.5	103	27	1	0.5

### Drug loading and release

#### Loading

**Scheme 1:**
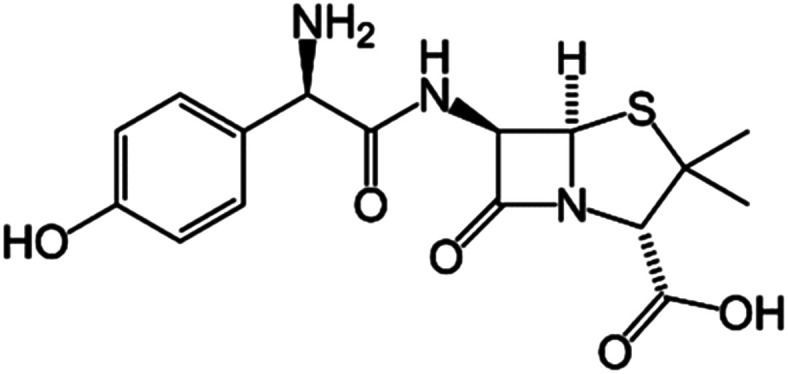
Chemical structure of an amoxicillin drug molecule.

Amoxicillin (Scheme 1) was chosen as the model drug molecule for the loading and release study of antibiotics. The study was performed by monitoring the changes of UV absorbance at the wavelength of 272 nm. The amoxicillin concentration was obtained through a standard curve that obeys the Beer–Lambert law. A series of aqueous solutions of amoxicillin with known concentrations (0.03–0.50 mg ml^−1^) was prepared and the absorbance was measured by using a Perkin-Elmer lambda 850 UV spectrometer.

For drug loading, 0.2 g of amino-functionalized glasses was dispersed in a 4 mg ml^−1^ aqueous solution of amoxicillin. The resulting solution was kept under continuous stirring for 24 hours. Then, bioactive glass loaded powders were collected by centrifugation at 10 000 rpm. The supernatants were analyzed using a UV spectrometer and the powders were dried at 60 °C for 24 hours. The loading efficiency of the three compositions was determined by using the following equation:1
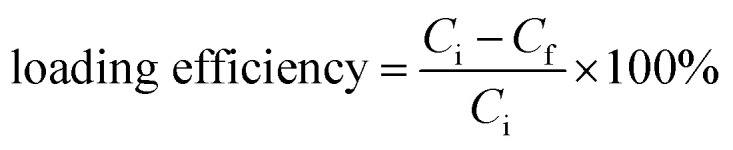
where *C*_i_ is the initial concentration of amoxicillin; *C*_f_ is the final concentration of the drug, which was calculated using the standard curve equation.

#### Drug release

An *in vitro* release study of amoxicillin was performed using phosphate buffered saline (pH = 7.4) as a release medium. Briefly, 0.1 g of the loaded bioactive glass powders was dispersed in 50 ml of PBS and kept under continuous stirring at 37 °C. After each selected time point, 4 ml was withdrawn and filtered, and another 4 ml of fresh PBS was added to the solution, keeping the total volume at 50 ml. The filtrated solution was analyzed by using UV-vis spectrometry and the concentration was calculated by using the standard equation. The cumulative release profile of amoxicillin in PBS was determined through the following equation:^30^2
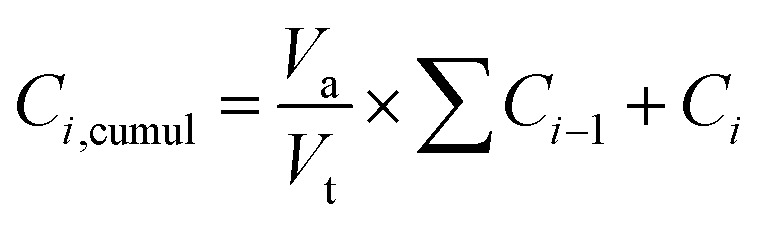
where *C*_*i*,cumul_ is the cumulative concentration; *C*_*i*_ is the concentration at time *i*; *C*_*i*−1_ is the concentration at time *i* − 1; *V*_a_ is the volume of the withdrawn solution and *V*_t_ is the volume of the total solution.

## Results and discussion

### Thermogravimetric analysis

**Fig. 1:**
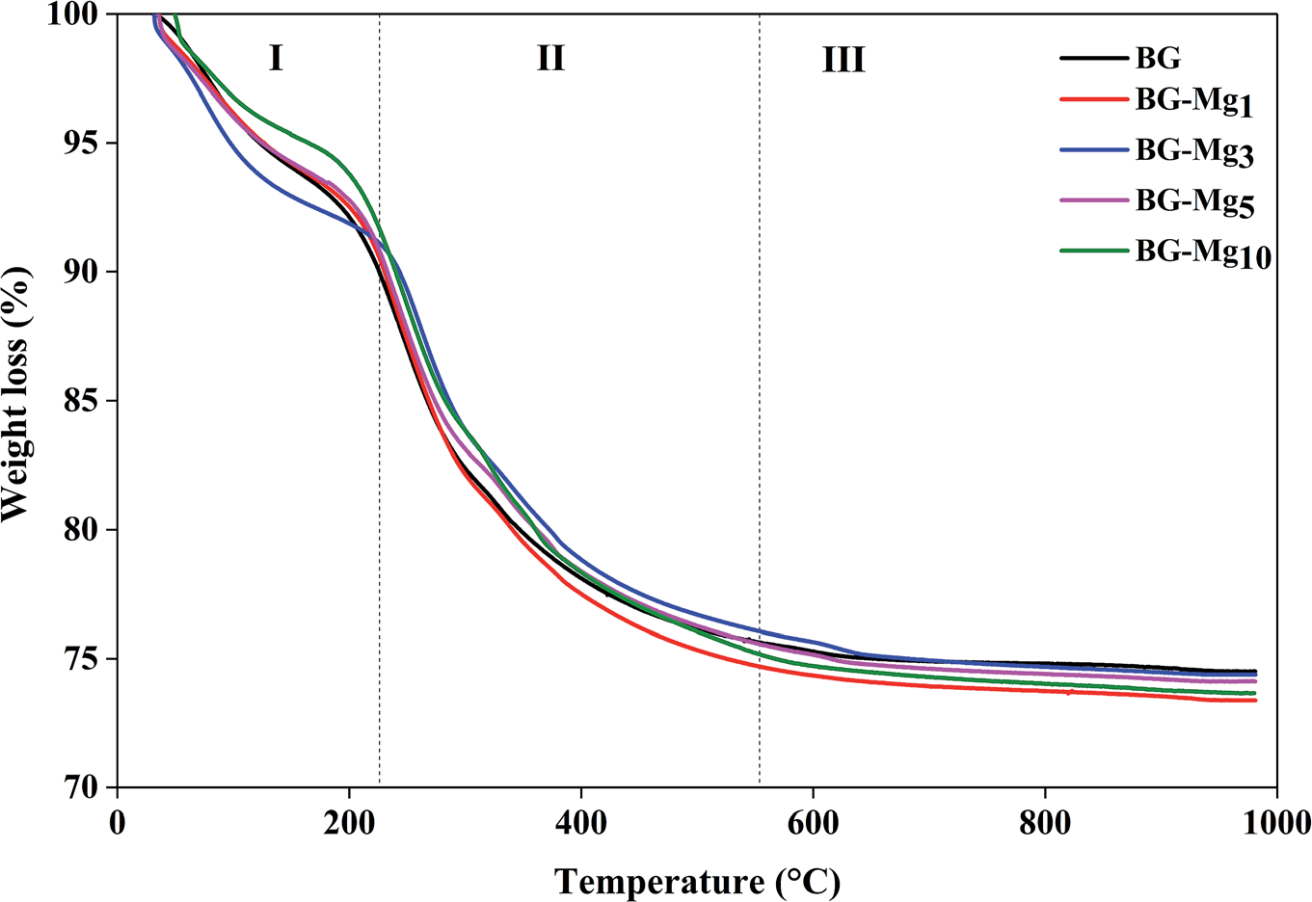
TGA profiles of bioactive glass powders.

Fig. 1 shows the TGA results of the as-dried powders. It was found that the total weight loss of the samples increases with MgO content after the heat treatment of the powders from room temperature to 1000 °C. The same observations were also noted by Mariappan C. R. *et al.* when they studied the influence of silver on the structure of bioglass-ceramic nanoparticles.^31^ The first range between 30 °C and 220 °C corresponds to the loss of physically adsorbed water molecules (from 30–120 °C) and condensation of silanol groups present at the surface of the nanoparticle samples (from 120–220 °C). For BG-Mg_10_, there was only a 4 wt% weight loss around 120 °C while BG-Mg_3_ recorded a significant weight loss of approximately 7 wt% in the same temperature range, mainly due to its ability to absorb more water molecules. In the range of 220–550 °C, a significant weight loss was noted for all samples, of about 14 wt%, which can be associated with the elimination of residual CTAB template, and residual nitrate and alkoxide groups that did not react during synthesis. Above 550 °C, the weight loss reached a steady state for all samples (73–75 wt%). In summary, all materials were heat treated at 550 °C before being used for the rest of this work.

### Composition

The results obtained through ICP-AES analysis are presented in Table 3 as an average of three measurements. The experimental composition of the sample was revealed to be close to the nominal composition. Silica content was found to be lower than the nominal value for all compositions. This might indicate that the amount of TEOS added during synthesis did not react totally and that the unreacted precursor molecules were most likely washed out during filtration. Overall, these results suggest the successful sol–gel synthesis of the present bioactive glass series.

**Table 3:** Molar compositions determined *via* ICP-AES

Sample acronym	Molar composition determined by ICP-AES		
	SiO_2_% mol	CaO% mol	MgO% mol
BG	77.08 ± 0.04	15.09 ± 0.06	0.0000
BG-Mg_1_	77.96 ± 0.02	14.77 ± 0.06	1.06 ± 0.05
BG-Mg_3_	77.48 ± 0.07	11.47 ± 0.04	2.50 ± 0.04
BG-Mg_5_	78.52 ± 0.05	9.95 ± 0.02	5.18 ± 0.03
BG-Mg_10_	78.24 ± 0.03	4.87 ± 0.04	10.64 ± 0.05

### Structural properties

**Fig. 2:**
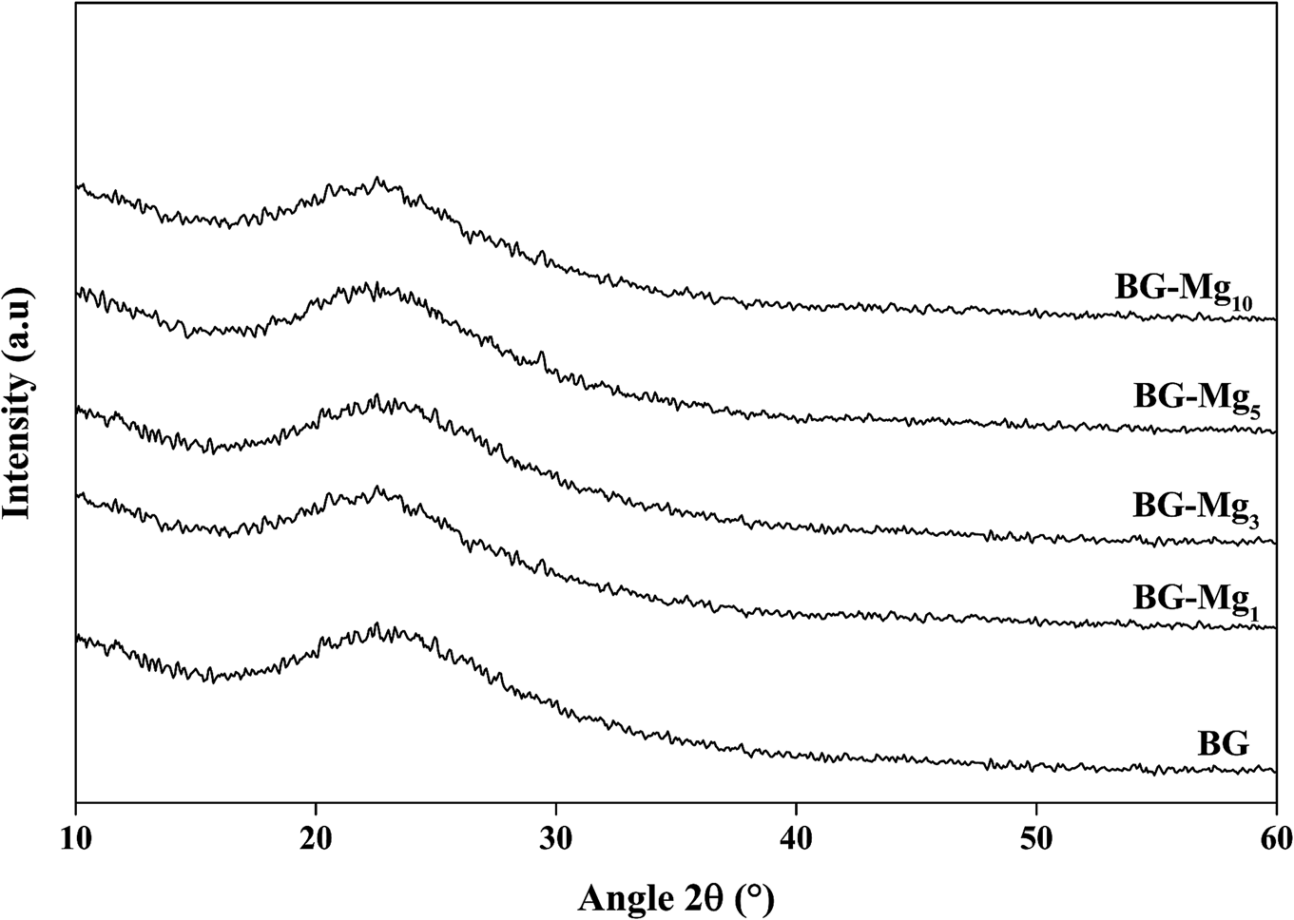
XRD patterns of the glasses after heat treatment at 550 °C.

Fig. 2 displays the XRD patterns for the glasses 85SiO_2_–(15 − *x*)CaO–*x*MgO (0 ≤ *x* ≤ 10) synthesized by the sol–gel route. All samples exhibited a broad peak between 15° and 30° after heat treatment at 550 °C. This indicates the absence of any crystalline phase and the presence of an entirely amorphous structure characteristic of the glassy phase, despite the increasing amount of magnesium. It is also important to point out that all five patterns are the same and did not contain diffraction peaks, revealing a disordered arrangement at the nanoscale level. This suggested that magnesium ions were successfully introduced into the glass structure. The same results were also obtained by El-Fiqi *et al.*^32^ and Riti *et al.*^24^

**Fig. 3:**
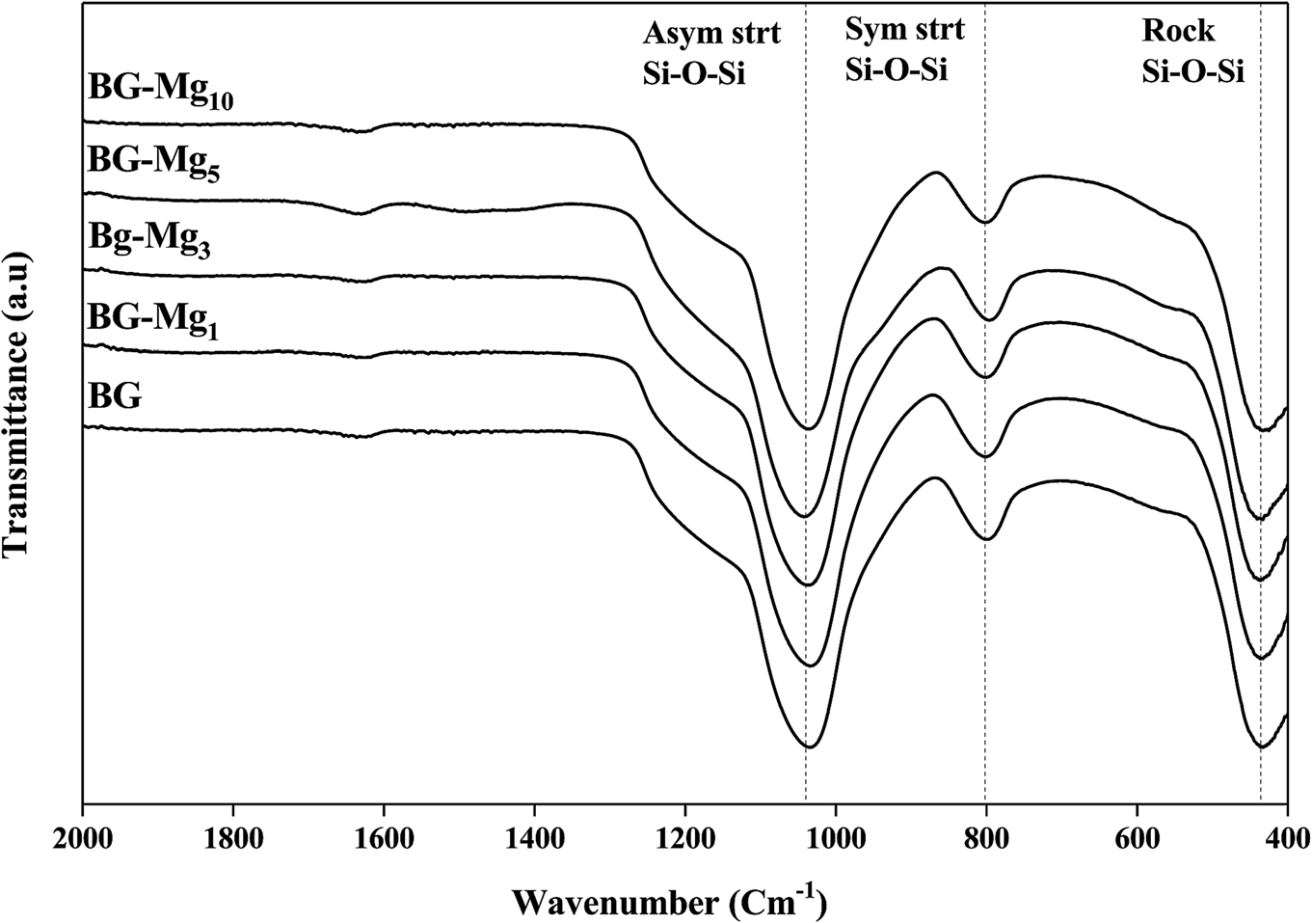
FTIR spectra of bioactive glass powders calcined at 550 °C.

The infrared absorption spectra of the five silicate materials after calcination at 550 °C are shown in Fig. 3. The results indicate the presence of three characteristic main bands allocated to different vibration modes of the Si–O–Si bonds.^33–35^ The first peak at 430 cm^−1^ corresponds to the rocking vibration of the Si–O–Si bending mode. The second band at 800 cm^−1^ was assigned to the symmetric stretching vibration of Si–O. It is worth noting that there is a shoulder at approximately 960 cm^−1^ before calcination (results not shown), which is related to the stretching vibration of Si–OH bonds, but it disappears totally after calcination, indicating the increase in network connectivity and the formation of more bridging oxygen.^35^ The third peak located at around 1030 cm^−1^ corresponds to the Si–O–Si asymmetric stretching mode, and it is the most pronounced in the spectrum of Mg free bioglass 85S15C. This band represents the silica network of BG-Mg_*x*_. The same bands were also observed in the work of Arcos *et al.*^36^ for mesoporous 85SiO_2_–10CaO–5P_2_O_5_ bioactive glass.

It is important to point out that a calcination temperature above 450 °C is necessary in order to successfully incorporate the Ca^2+^ and Mg^2+^ ions into the glass structure.^37–39^ FTIR spectra and XRD patterns of the different bioactive glass compositions did not show major differences. In this work, the total modifier molar percentage is equal to 15% in all compositions. For this reason, we believe that only minor changes could be detected through FTIR and XRD analysis. Galiano *et al.*^40^ studied Mg and Sr incorporation in the glass network. In this study, the authors found that alkaline earth metals do not have an influence on the distribution of the Qn species in the silica network as long as the total RO fraction did not change, where R = Ca, Mg, Sr. This also confirms why the FTIR spectra of different glasses recorded in this work did not show much of a difference. The same conclusions were obtained by Pedone *et al.*,^41^ where the short range distribution of the Qn species did not change when varying MgO content in the glass composition. These results support our assumptions on the structural analysis of our glass powders with different CaO/MgO ratios.

### Textural properties

**Fig. 4:**
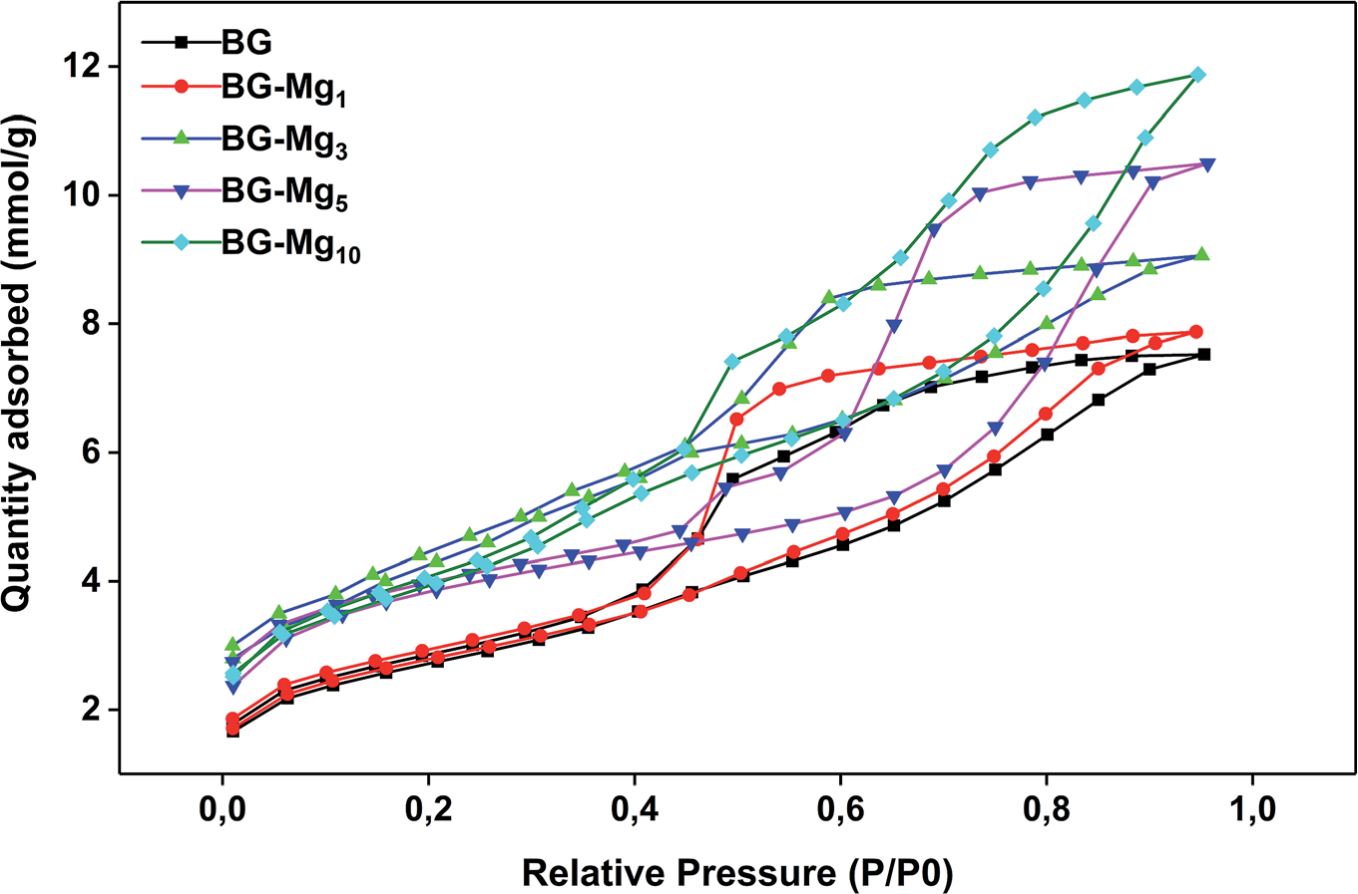
N_2_ adsorption–desorption isotherms for all studied glasses.

N_2_ adsorption isotherm analyses were performed to determine the textural properties of the bioactive glasses in terms of surface area, pore volume and pore size distribution. Fig. 4 shows the adsorption/desorption isotherms obtained for various compositions. According to the recent update of the IUPAC classification,^42^ all samples revealed a type (IVa) isotherm with a hysteresis loop between 0.4 and 0.9 *P*/*P*_0_. The characteristic hysteresis loop is observed in all cases, which evidenced the presence of mesopores in the glasses. However, there are noticeable differences in the shape of the hysteresis. As can be seen, the closing point of the hysteresis, the *P*/*P*_0_ pressure at which the desorption branch joins the adsorption branch, shifts to higher values as the magnesium content in the glasses increased. For BG, BG-Mg_5_ and BG-Mg_10_, an isotherm with type H5 hysteresis was observed. This suggests that these three compositions contain both open and partially blocked mesopores. Concerning BG-Mg_1_ and BG-Mg_3_, a hysteresis of type H2(a) was observed, which indicates the existence of ink-bottle shaped pores. Examples of this type of hysteresis loop have been observed with mesocellular silica foams and certain mesoporous ordered silica after hydrothermal treatment.^42^

Surface area was determined by means of the BET method. According to the calculations, surface area increases with increasing magnesium content in the glass composition, as indicated in Table 4. As can be evidenced, the surface area increased from 211 to 311 m^2^ g^−1^, and the pore volume increased from 0.2616 to 0.4127 cm^3^ g^−1^, when going from BG to BG-Mg_10_. Our results are similar to those found by Li *et al.*,^43^ in which surface area increased with increasing Mg molar substitution of Ca in the system Si–Ca–P–Mg.

**Table 4:** Textural properties of the examined glasses

Composition	Surface area m^2^ g^−1^	Total pore volume cm^3^ g^−1^	Average pore size nm	Average particle size nm
BG	211.6407	0.2616	5.1598	28.3499
BG-Mg_1_	215.1095	0.2736	5.3753	27.8928
BG-Mg_3_	250.3050	0.3090	5.3887	25.1997
BG-Mg_5_	284.2020	0.3649	5.4366	21.1117
BG-Mg_10_	311.9174	0.4127	5.4933	19.2359

### Bioactivity assessment

The essential sign of bioactivity is the ability to form an apatite layer on the glass surface that chemically bridges the bone and the implant. To determine the bioactivity of the synthesized glasses, they were subjected to *in vitro* solution testing using SBF medium. The samples were soaked in SBF at 37 °C for different periods of time from 1 hour to 14 days. After each time period, the solutions were filtered, their pH was measured and the powders were analyzed using FTIR spectroscopy, XRD and SEM.

**Fig. 5:**
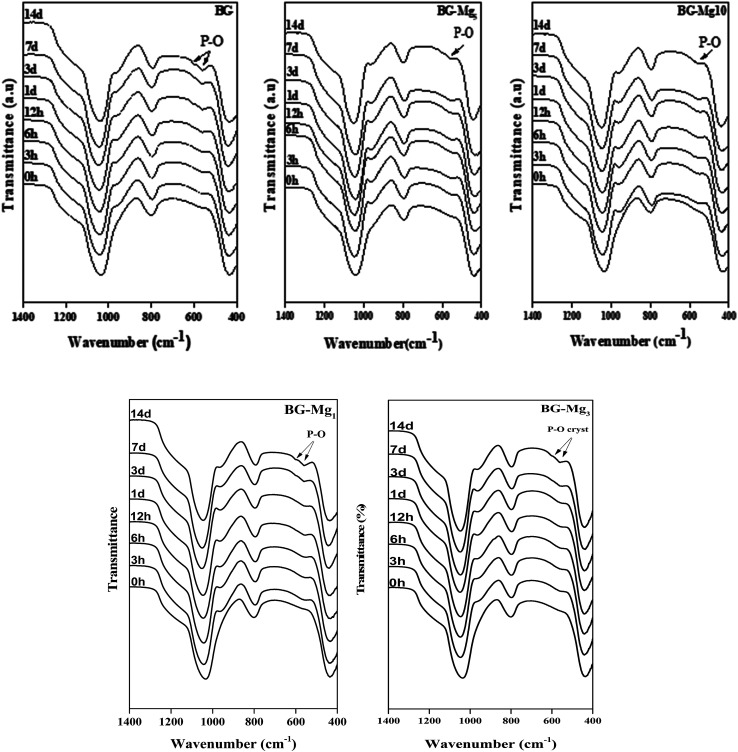
FTIR spectra of BG0, BG-Mg_*x*_ after each period in the bioactivity test.

**Fig. 6:**
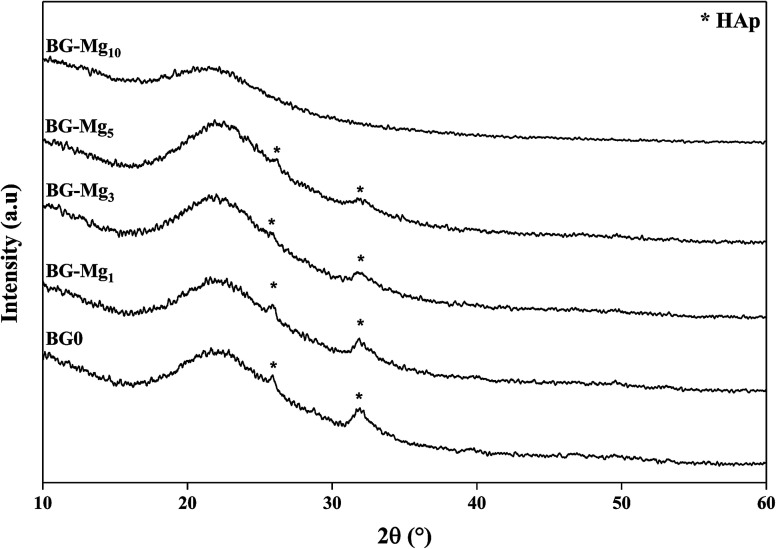
XRD patterns of the BG-Mg series after 14 days of immersion in SBF.

Fig. 5 shows the FTIR spectra of the samples after soaking in SBF for different periods of time. The comparison between the spectra before and after immersion in SBF displays the appearance of additional peaks around 500 cm^−1^ after only 3 hours of testing for all studied glasses,^34^ related to the antisymmetric vibration mode of P–O in an amorphous Ca–P layer, which indicate the formation of apatite in SBF. In addition, after 3 days of soaking for BG, BG-Mg_1_ and BG-Mg_3_ (data not shown), the spectra exhibit absorption bands at 560 and 604 cm^−1^, corresponding to the P–O vibrational mode in a crystalline phosphate phase.^14,31^ However, BG-Mg_5_ and BG-Mg_10_ powders did not exhibit any crystallization of the amorphous Ca–P already deposited after only 3 hours of immersion. These results led us to suggest that Mg incorporation in the glass composition has a retarding effect on the crystallization of the Ca–P amorphous layer deposited in the early stages of the bioactivity test. To further confirm our assumption, XRD analysis was conducted after 14 days in SBF. The XRD patterns of samples after 14 days in SBF are displayed in Fig. 6. These patterns show the broad band characteristic of internal disorder and the glassy nature of these materials. However, new wide reflection peaks can be detected for some samples, indicating the formation of a poorly crystallized hydroxyapatite phase. For BG and BG-Mg_*x*_ (*x* = 1, 3, 5), the XRD patterns indicate the presence of two additional peaks at 25° and 32°, which can be ascribed to diffraction of the (002) and (211) planes in a poorly crystalline apatite phase,^44^ respectively. For BG-Mg_3_ and BG-Mg_5_, the peak situated at 32° becomes wider while the intensity of the peak situated at 25° showed a reduction in its intensity. In the case of BG-Mg_10_, no sign of a crystalline phase could be observed. These findings are in good accordance with the FTIR observations.

**Fig. 7:**
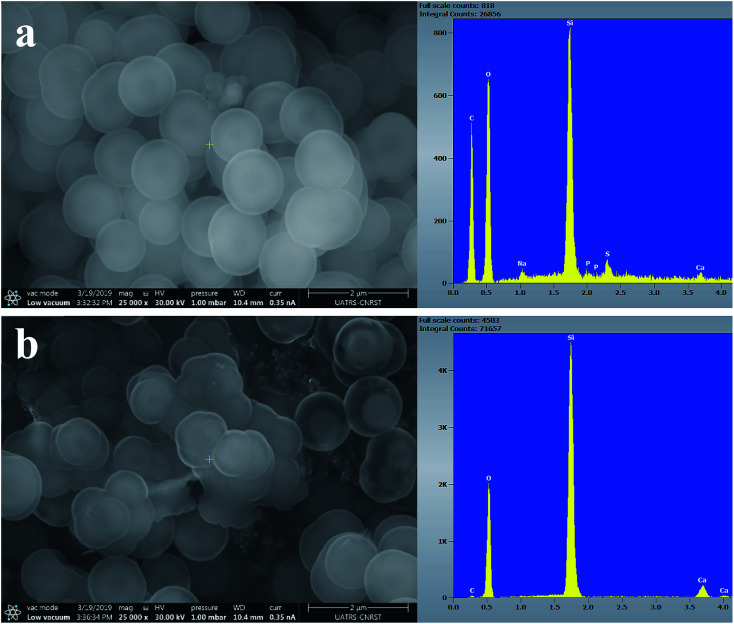
SEM images of BG0 (a) before and (b) after 14 days of immersion in SBF.

**Fig. 8:**
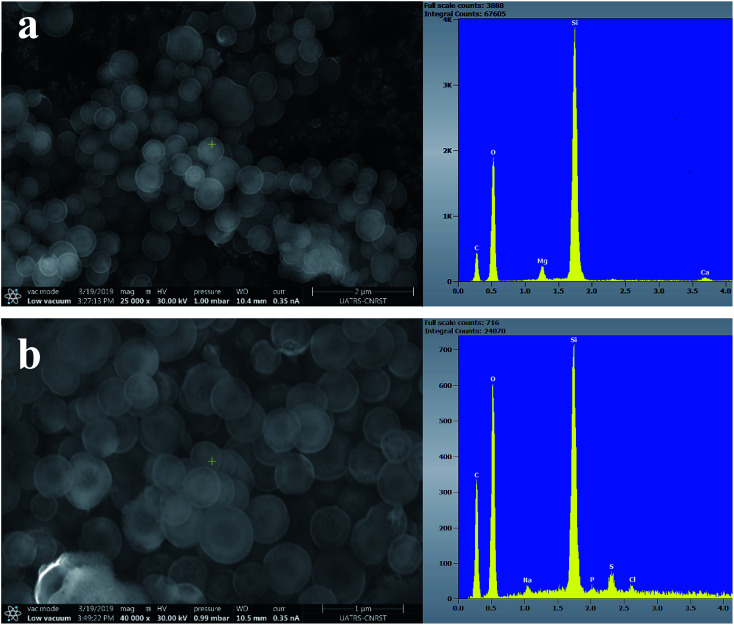
SEM images of BG-Mg_3_ (a) before and (b) after 14 days of immersion in SBF.

**Fig. 9:**
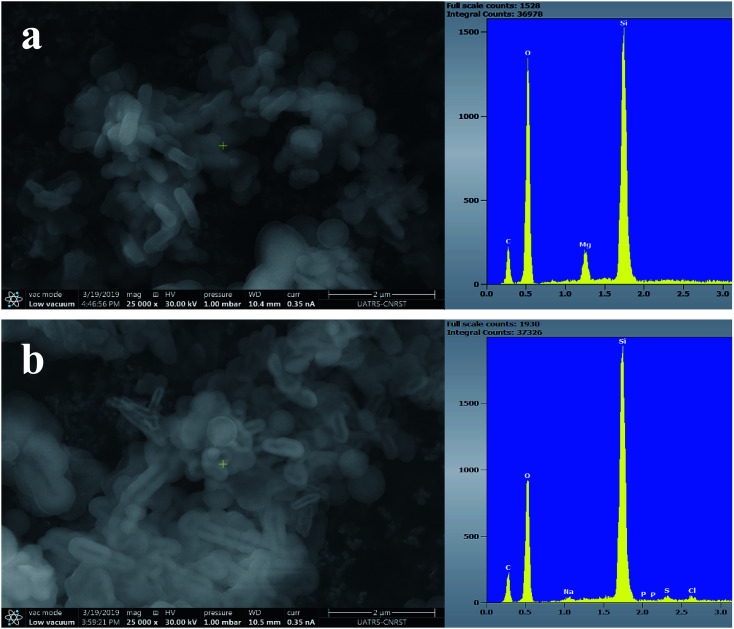
SEM images of BG-Mg_5_ (a) before and (b) after 14 days of immersion in SBF.

Fig. 7–9 show the SEM images and EDS spectra of BG, BG-Mg_3_ and BG-Mg_5_ before and after 14 days of immersion in SBF. Before soaking, the micrographs show particles with spherical and regular shapes. A slight decrease in particle size was also noticed when the amount of magnesium was increased, see Fig. 7, 8 and 9(a). EDS analysis of the bioactive glass powder confirmed the presence of Mg and Ca elements in the glass nanoparticles. Comparing the EDS results with the theoretical values of the elemental composition of our bioactive glass nanoparticles, it was found that a gap exists between the sets of data. However, the EDS values were obtained only for the surface. It is hypothesized that the distribution of cations in the glass nanoparticles is not uniform. This difference in composition may also originate from the washing step during synthesis, which leads to the loss of unreacted chemicals along with poorly adhered cations on the surface, since the modifier cations such as calcium are present on the surface of the glass particles and do not enter the glass structure until heat treatment.

After 14 days in SBF (Fig. 7, 8 and 9(b)), it was observed that for the BG composition, the nanoparticle formation appeared to be impeded with the newly formed layer of small Ca–P crystallites, which confirms their bioactivity, as was also reported by other research groups.^11,45,46^ In the case of BG-Mg_3_ and BG-Mg_5_, the SEM images did not present the globular shape frequently found for apatite, but only showed that the particles had agglomerated, indicating the formation of an accentuated pseudo-crystalline calcium phosphate layer on their surfaces and between the particles. These results are consistent with the FTIR and XRD observations. All these findings indicated that magnesium has a retarding effect on the bioactivity of the glass and more precisely on the crystallization of the Ca–P amorphous layer. Works done by other research groups showed the same retarding effect of Mg on glass bioactivity.^22^ It is supposed that Mg may enter the formed nuclei of hydroxyapatite, which results in changing its physico-chemical properties and thus hindering its growth.^47^ It was also reported that Mg favors the formation of amorphous calcium phosphate and suppresses its crystallization when it is present at high concentrations.^48^

**Fig. 10:**
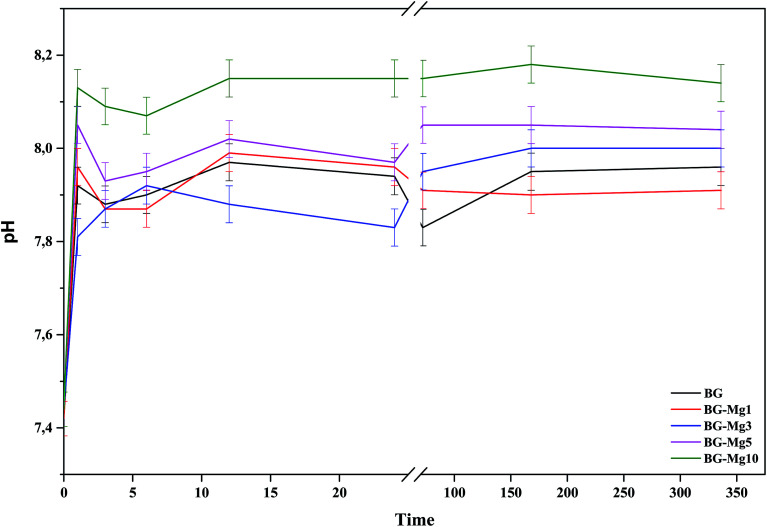
pH variations during the bioactivity tests in SBF of the BG-Mg samples.

The variation of pH in SBF medium as a function of time for BG, BG-Mg_1_, BG-Mg_3_, BG-Mg_5_ and BG-Mg_10_ is presented in Fig. 10. As can be seen, the five samples exhibited almost the same pH variation profile with a perpendicular trend during the first hour followed by a slight decrease at 3 h then a stabilization of pH values until the end of the test. The same changes were detected by Balamurugan *et al.*^49^

In a general way, for the first few hours of the test, a rapid dissolution of the glass surface occurs, which is accompanied by an ionic exchange between the Ca^2+^ and Mg^2+^ ions from the glass and H^+^ ions present in the SBF. Moreover, the variation of pH depends closely on Mg content in the bioactive glass compositions.^50^ pH values at one hour of immersion in SBF for BG, BG-Mg_5_ and BG-Mg_10_ are 7.92, 8.05 and 8.13, respectively. This suggest that glasses with a higher Mg content are more prone to dissolution.^51^ This is in contrast to other research,^50,52^ in which the authors found that Mg incorporation tends to slow down the glass dissolution when in contact with a biological medium. Our assumption can be explained by the fact that glasses doped with high magnesium fractions have higher surface areas than those with no, or lower Mg content. With higher surface areas, bioactive glasses are more prone to dissolution and to have a more intense ionic exchange between their surface and the surrounding medium. As a result, there is a significant increase of the pH of the SBF solutions.

L. Hench^5^ proposed that the mechanism of bioactivity involves five steps: glass dissolution and ionic exchange between the glass surface and the biological medium, repolymerization of a silica rich layer on the surface, formation of an amorphous Ca–P layer and finally the crystallization of this layer into a carbonated hydroxyapatite (CHA). It is worth mentioning that hydroxyapatite is the most stable phase of calcium phosphates.^53^ Therefore, the ups and down observed in the pH variation profiles during the first few hours of the test could be related to the dissolution of a less stable Ca–P phase until its final stabilization.^50^ Additionally, Mg doped hydroxyapatite has been shown to have a faster dissolution rate.^54^ This was explained by the fact that Mg^2+^ has a smaller radius compared to Ca^2+^ (0.72 and 1.00, respectively), which creates voids between particles.

### Kinetics of drug delivery

**Fig. 11:**
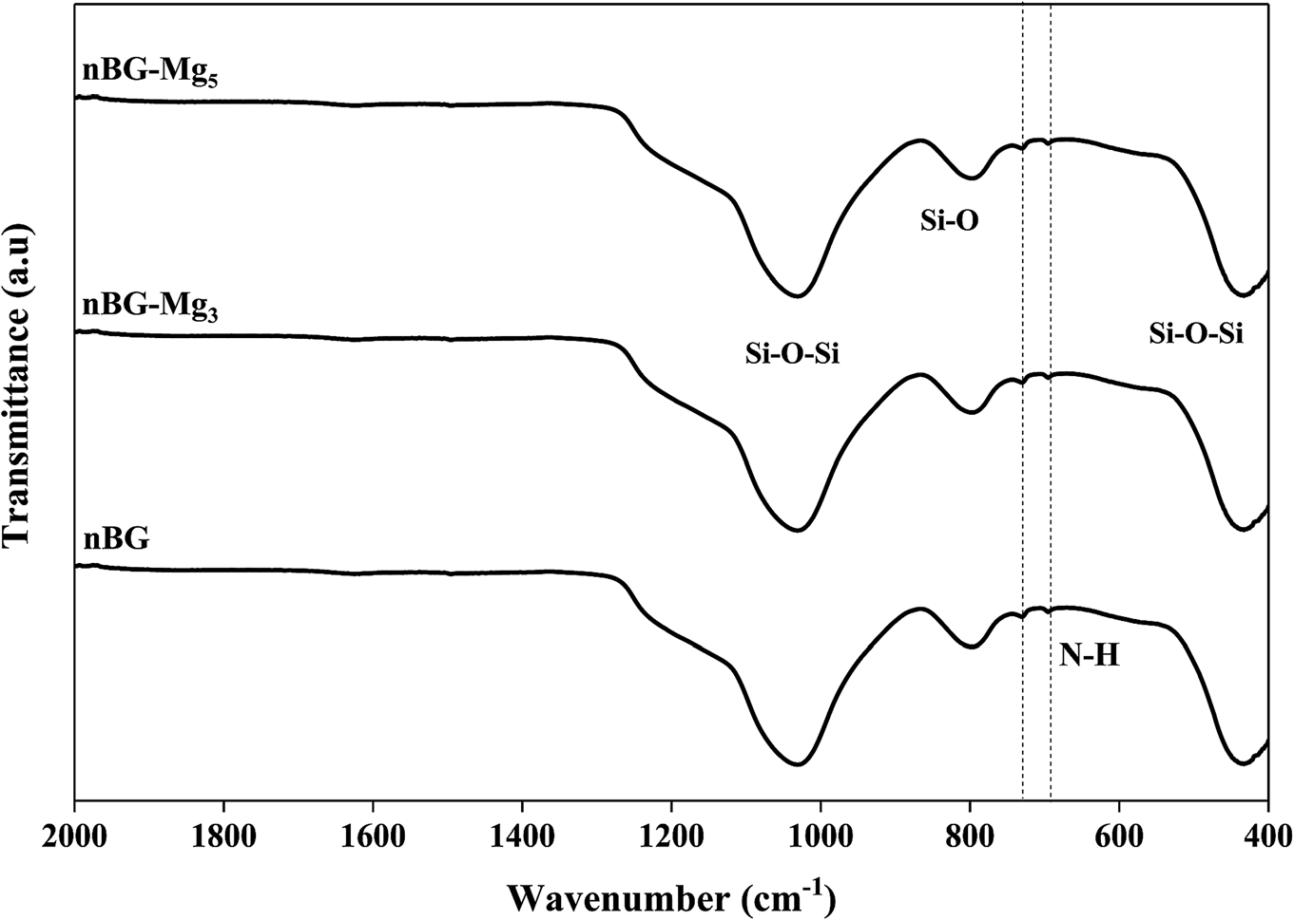
FTIR spectra of BG, BG-Mg_3_ and Bg-Mg_5_ samples after amine functionalization.

Bioactive glass nanoparticles are rich with hydroxide (OH) groups on their surface, which confer to them a net negative surface charge. This negatively charged surface is considered as a major hurdle in loading negatively charged biomolecules such as amoxicillin.^55^ To overcome this obstacle, the surface of the glass particles was modified. For this purpose, surface amination was used. It was hypothesized that after functionalization, the surface status would change from hydroxyl groups to amino groups, and thus the behavior of our nanoparticles would change when interacting with biomolecules of a negative charge nature. During biomineralization in SBF, it was shown that n-BG, n-BG-Mg_3_ and n-BG-Mg_5_ presented a pseudocrystalline structure after 14 days of immersion, and therefore they were chosen for drug release testing. The FTIR spectra of the samples aminated with APTES are shown in Fig. 11. The spectra reveal the presence of two additional peaks at 730 and 695 cm^−1^ compared to the non-aminated powders whose spectra present only the characteristic bands of Si–O–Si and Si–O functional groups, as shown in Fig. 4. These new peaks are related to the vibration mode of the N–H bond in the amino group.^26^ This result suggests the successful functionalization of the bioactive glasses. The presence of amino groups instead of hydroxyl groups on the bioactive glass surfaces is supposed to help in increasing the drug loading capacity of our glass powders since there will be more sites of interaction between the drug and the functionalized surface.^56^

**Fig. 12:**
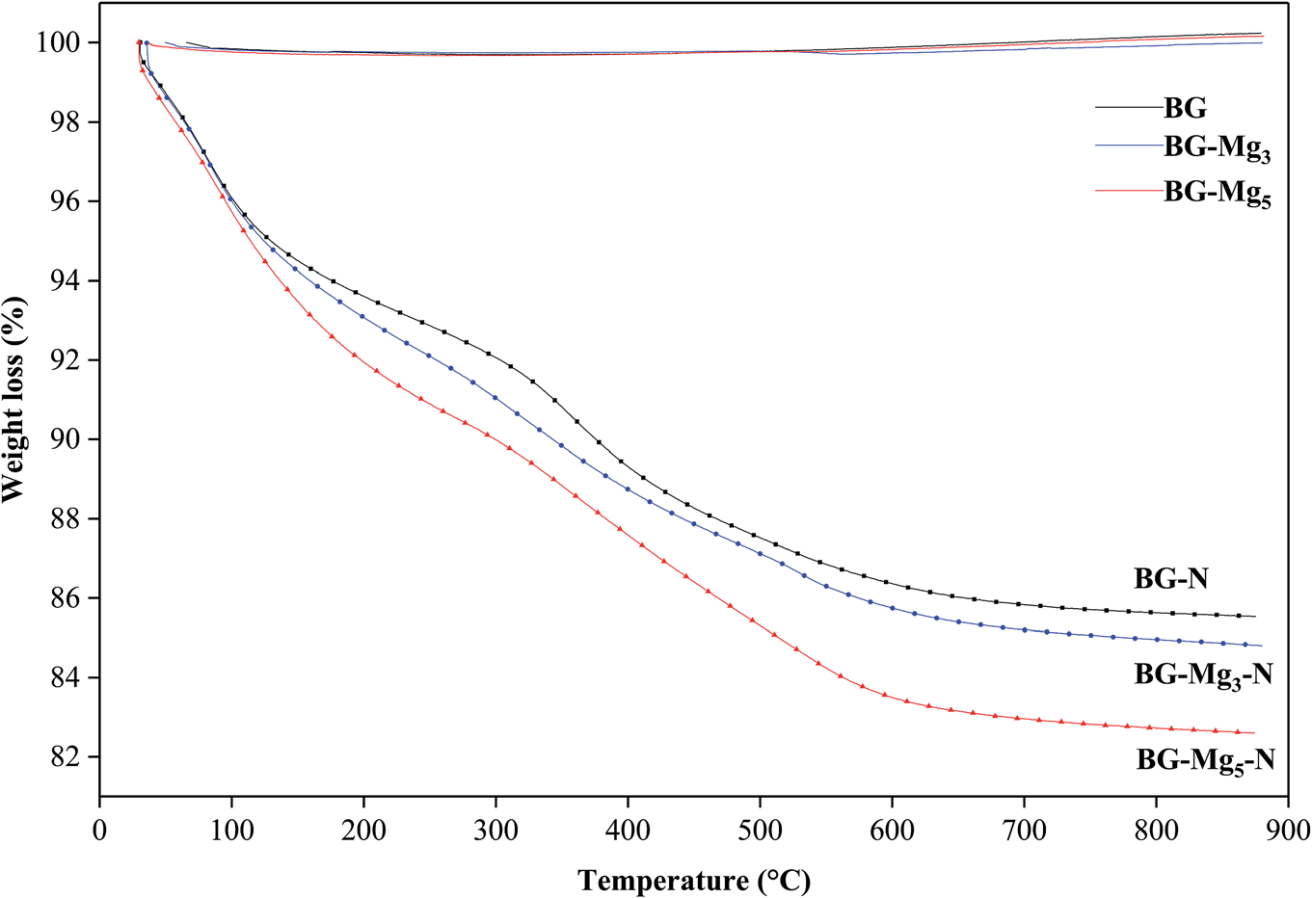
TGA spectra of bioglasses heat treated at 550 °C without and with APTES loading.

In order to figure out the relationship between surface area and the amount of APTES grafted on the bioglasses, TGA tests were performed. As can be deduced from Fig. 12, the amount of APTES increased with increasing surface area from n-BG to n-BG-Mg_5_, as can be seen by the total weight loss around 850 °C. According to the data, it was also observed that there are three stages of weight loss. The first one corresponded to the removal of physically adsorbed water (until 200 °C). More weight loss started from the end of the first weight loss stage (200 °C) until about 470 °C and was most likely due to the loss of organics (*i.e.* alkoxy groups). The third drop in mass occurred from the end of the second weight loss (470 °C) until around 600 °C, corresponding to the residual amino groups introduced by APTES during the grafting procedure.

**Fig. 13:**
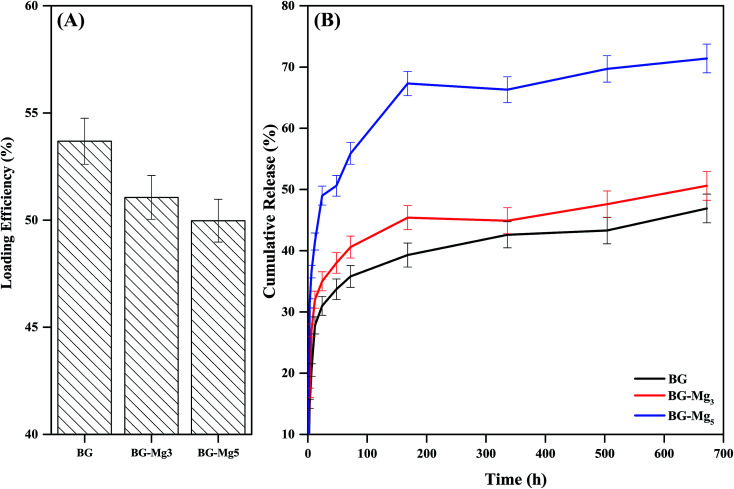
Loading efficiency (A) of BG, BG-Mg_3_ and BG-Mg_5_ and their release profiles (B) of amoxicillin in PBS.

Drug loading was performed in an amoxicillin aqueous solution of 4 mg ml^−1^. The results shown in Fig. 13A indicate that the drug loading efficiency slightly decreased with an increase of Mg content in the glass composition. n-BG had the highest loading efficiency with 53.68% of amoxicillin loaded, followed by n-BG-Mg_3_ and n-BG-Mg_5_ with 51.06% and 49.97%, respectively. Drug loading efficiency is influenced by 4 major parameters: pore size, pore volume, surface area and drug–glass interaction.^51,57^ Pore size should be high enough to permit incorporation of drug molecules into the pore network. In this sense, a minimum ratio of pore size/drug molecular size of 1 is recommended.^51^ In the present study, the average pore size was found to be in the range of 5–6 nm, which is larger than the molecular length of amoxicillin of 1.1 nm.^57^ Furthermore, the loading results obtained in the present research are superior to those already reported in the literature of adsorption of amoxicillin into silica based mesoporous matrices.^7,8,58^ One possible explanation for the high amounts of drug loaded into our bioactive glasses is the amino functionalization. Drug–material interaction is one of the major parameters to be considered and the selection of the functionalization group must be done considering the targeted molecule.^59^ It was reported that drug loading could be enhanced with aminopropyl, compared with other groups,^58^ due to its high affinity to the carboxyl groups present in amoxicillin.^60^ In addition to the stronger bond of COO^−^–NH_3_^+^ than that of COO^−^–OH,^61^ this would give more sites of interaction and thus more drug molecules to be adsorbed onto the surface of our powders. The slight difference observed in our case is related to the geometry of the pores in each composition. For the virgin glasses, a slightly higher value was recorded compared to the Mg doped glasses. This is probably due to the cylindrical pore geometry that allowed no more APTES molecules to be loaded between the grafted APTES on the glass surfaces and this relatively enhanced AMX adsorption on the surface. Where pore size was increased (BG-Mg_3_ and BG-Mg_5_), additional APTES molecules could be inserted into the ink bottle shaped pores, which limited the accessibility of the AMX drug molecules for adsorption, and the drug molecules were content to segregate mainly on the surface of the glasses.

Drug release was performed in PBS (pH = 7.4) at 37 °C and monitored using UV-spectroscopy. Drug release profiles of amoxicillin loaded n-BG, n-BG-Mg_3_ and n-BG-Mg_5_ are shown in Fig. 13B. As can be seen, all release profiles follow a first-order release kinetics, characteristic of a diffusion process.^51^ All three compositions show the same release profile. A quick release^62^ in the first 7 days is followed by a decrease in the rate of release that finally stabilizes. A burst release is desirable, especially where a high concentration of drug is needed; for example, during the first days of implantation in a bone defect, when a high risk of inflammatory responses and infection is present.

The difference between the three profiles lies in the increase of the cumulative amount of drug delivered with increasing magnesium content, which in turn is related to the increase of the specific surface area and the total pore volume of the bioglass powders. This means that the release kinetics of amoxicillin increased although the total amount of drug loaded decreased.

The TGA data after functionalization led us to conclude that the number of amino groups grafted on the surface of the bioactive glasses increased with increasing magnesium content in the glass composition due to the higher textural properties. This would result in the filling of the pores, which suggests that loading of amoxicillin on BG-Mg_5_ took place primarily on the surface. Our assumption also explains the difference in the drug release kinetics between the three compositions. The surface loaded molecules of amoxicillin would be easily released to the medium, in contrast to the drug molecules trapped inside the pores.

## Conclusions

Mesoporous bioactive glass nanoparticles doped with magnesium were synthetized using a sol–gel method. Primary characterization showed that the nanoparticles had a spherical form and a pore distribution in the meso range (2–50 nm). The *in vitro* study of their bioactivity revealed that Mg incorporation at high molar fractions into the glass network resulted in a fast dissolution rate and solubility of the glass, and a retarding effect on the crystallization of the amorphous Ca–P layer. Further studies of amoxicillin loading and release led to two additional conclusions. Loading efficiency decreased with increasing magnesium content, whereas the release kinetics, which followed a first order profile, increased. The 85SiO_2_–10CaO–5MgO composition showed better results, which was suggested to be due to its higher surface area and pore size. The porosity, surface area and chemical composition of the bioactive glasses played a significant role in controlling the bioactive behavior of the glass samples.

## Conflicts of interest

There are no conflicts to declare.

## Supplementary Material
